# Geodetic and Remote-Sensing Sensors for Dam Deformation Monitoring

**DOI:** 10.3390/s18113682

**Published:** 2018-10-29

**Authors:** Marco Scaioni, Maria Marsella, Michele Crosetto, Vincenza Tornatore, Jin Wang

**Affiliations:** 1 Department of Architecture, Built Environment and Construction Engineering (DABC), Politecnico di Milano, 20133 Milano, Italy; marco.scaioni@polimi.it; 2 College of Surveying and Geo-Informatics, Tongji University, Shanghai 200092, China; 3 Department of Civil, Environmental and Building Engineering (DICEA), Università degli Studi di Roma ‘La Sapienza’, 00184 Roma, Italy; 4 Division of Geomatics, Centre Tecnològic de Telecomunicacions de Catalunya (CTTC/CERCA), E-08860 Castelldefels, Barcelona, Spain; mcrosetto@cttc.cat; 5 Department of Civil and Environmental Engineering (DICA), Politecnico di Milano, 20133 Milano, Italy; vincenza.tornatore@polimi.it; 6 Beijing Key Laboratory of Traffic Engineering, Beijing University of Technology, Beijing 100124, China; wangjin.smile@hotmail.com

**Keywords:** dams, deformation measurement, D-InSAR, GNSS, ground-based SAR, integrated monitoring systems, terrestrial laser scanning

## Abstract

In recent years, the measurement of dam displacements has benefited from a great improvement of existing technology, which has allowed a higher degree of automation. This has led to data collection with an improved temporal and spatial resolution. Robotic total stations and GNSS (Global Navigation Satellite System) techniques, often in an integrated manner, may provide efficient solutions for measuring 3D displacements on precise locations on the outer surfaces of dams. On the other hand, remote-sensing techniques, such as terrestrial laser scanning, ground-based SAR (synthetic aperture radar) and satellite differential interferometric SAR offer the chance to extend the observed region to a large portion of a structure and its surrounding areas, integrating the information that is usually provided in a limited number of in-situ control points. The design and implementation of integrated monitoring systems have been revealed as a strategic solution to analyze different situations in a spatial and temporal context. Research devoted to the optimization of data processing tools has evolved with the aim of improving the accuracy and reliability of the measured deformations. The analysis of the observed data for the interpretation and prediction of dam deformations under external loads has been largely investigated on the basis of purely statistical or deterministic methods. The latter may integrate observation from geodetic, remote-sensing and geotechnical/structural sensors with mechanical models of the dam structure. In this paper, a review of the available technologies for dam deformation monitoring is provided, including those sensors that are already applied in routinary operations and some experimental solutions. The aim was to support people who are working in this field to have a complete view of existing solutions, as well as to understand future directions and trends.

## 1. Introduction

Monitoring the health of dam infrastructures has a key role in ensuring their safety conditions and maintaining their operational functions. Dam failures represent a high-risk for people, human settlements, infrastructures, and the environment. As a consequence, careful surveillance with the aim of detecting any possible critical situations is required. In addition, when the failure status is not completely reached but the operational conditions of a barrage cannot be fully guaranteed, a severe economic loss may result from to the interruption of energy production or other related activities such as hydraulic regulation and water storage.

The complexity of dams calls for the use of multiple sensors for monitoring. Each sensor focuses on a different area of the main barrage, the slopes surrounding the water reservoir, and the utility structures. In addition, different processes (structural deformations, water seepage, corrosion, and weathering) have to be measured using a suitable data acquisition rate that should be comparable to the velocity of the observed ongoing processes. Monitoring has not only the purpose of pre-alerting a forthcoming collapse but may provide useful information to verify the design parameters, to investigate the causative reasons of deformation processes, and to learn lessons to be implemented in future projects [1].

Measuring structural deformations of dams aims to detect rigid and non-rigid changes of the geometric shape of the structure, at both local and general level [2]. During its lifetime, a structure is subject to short- (daily/weekly/monthly) or long-term (years/decades) deformation processes, which may alter the original safety conditions. In order to set up an efficient deformation monitoring plan, the degradation level of the structure should be evaluated by using suitable sensors able to measure absolute and relative displacements of the dam. Usually, measurements are made on a limited number of control points (CPs), properly established in key positions to extrapolate the behavior of the whole structure. The deformation monitoring plan may be designed to analyze the long-term pattern [3,4] or may follow a specific event (e.g., an earthquake, as reported in Radhakrishnan [5]). The observation of dynamic movements requires high-frequency sensors [6] able to continuously track accelerations at specific points [7,8] or experimental global approaches [9].

Until the 1950s, the static monitoring of dams was mainly based on geodetic control networks [10,11] to measure absolute and relative displacements of the structure and the nearby areas (e.g., rock shoulders and slopes of the water basin). Despite the achievable precision, the related operations were quite complex and required a team of expert surveyors to work for a few days per each campaign. Geodetic networks were complemented by geotechnical/structural sensors [12] able to measure local deformations (e.g., tiltmeters, extensometers, strainmeters, clinometres) and other physical quantities (e.g., piezometers, load cells, stress cells). 

In the 1960s the introduction of automatic data acquisition and telemetric transmission allowed for the collection of data at a higher rate, up to continuous monitoring. These solutions provided long-term data series that improved the capability of analyzing deformation patterns. 

Over the years, technological developments have continuously increased the precision, the degree of automation, the data handling capability of the adopted geodetic and geotechnical sensor technology. In parallel, during the last decades, dam monitoring has benefited from the development of remote-sensing techniques from ground-based and satellite platforms. These have offered unprecedented opportunities for improving the structural analysis, since they extended the monitoring to a large portion of a structure, instead of a limited number of few CPs [13]. Among these techniques for areal deformation measurement (ADM), two types of ground-based sensors are included: terrestrial laser scanning [14] and ground-based SAR (synthetic aperture radar [15]). Differential interferometric SAR (D-InSAR) may help monitor displacements of the upper parts of large dams that are illuminated by spaceborne microwave sensors [16,17]. Since the first experimental applications of these methods, many improvements have been made and the current state-of-the-art features a much greater potential for field operations, including the capability of building up long time-series of observations (i.e., displacements in range direction between the sensor and the illuminated point). Such data sets can also be used for retrospective analyses using archives of SAR data (see, e.g., Milillo [18]).

Methods based on Global Navigation Satellite Systems (GNSS) have been widely applied for the measurement of the dam’s displacements and in the nearby areas. Initially, GNSS networks were adopted for periodic measurements of CPs, controlled by a set of reference points established in proximal stable areas. In the recent years, automatic systems adopting differential GNSS sensors, able to work in continuous mode, have been developed to be integrated into early-warning systems for the safe maintenance of the dam [19,20]. Real-time kinematic (RTK) measurements have also been used to carry out high-accuracy dam deformation monitoring [21,22].

Since there is not any sensor capable of recording the full information needed for monitoring a dam, great attention has been concentrated on the development of integrated monitoring systems (IMS), see Chrzanowski [1]. Moreover, the methodologies for data processing and integration have improved for analyzing different observations in a wider spatial and temporal context. Therefore, deformation patterns can be better understood by merging and cross-validating multiple observations. Validation with respect to ground-based sensors is particularly relevant when satellite-based techniques are adopted (e.g., D-InSAR). Monitoring data can be used to investigate correlations with external loads (e.g., water level in the reservoir and external temperature) by using statistical or deterministic methods [1]. Recent research trends have been moving towards the integration of monitoring observations and structural modeling [16,23].

This paper provides an overview of existing and innovative geodetic and remote-sensing methods for dam deformation monitoring. A description of the characteristics of deformations that can be observed on a dam is given in Section 2. Sensor technologies are illustrated on the basis of a classification in two main classes: (i) Geodetic and GNSS sensors, which may provide precise measurements at specific locations (Section 3) and (ii) ground-based/spaceborne remote sensors, which may output distributed measurements (ADM) over a large area (Section 4). The analysis of the main innovations in data processing and sensor integration/data fusion techniques (Section 5) precedes the final discussion on the monitoring techniques and on the future perspectives in dam deformation monitoring (Section 6).

## 2. Which Are the Types of Deformations to be Measured in Dams?

Dams are generally classified as earth-filled [17], concrete face rock-filled [24], and concrete [25] structures that, clearly, react differently to external loads. Concrete structures can be then being categorized into two main groups depending on the static principle: gravity dams, single and double curvature dams.

The external inputs that may alter the geometry of a dam can be ordinary, including thermal forces due to the air temperature and solar warming [26], and hydrostatic water pressure from the basin [27]. Extraordinary causative reasons mainly include seismic events [28,29] and the alteration of geotechnical conditions of the substrate [30]. On the other hand, inner phenomena may also result in deformations, such as the change in the strain-stress curve of the structural materials (e.g., due to plasticity processes), local sinking or sliding of the dam body with respect to the bedrock, and water sea-page in earth-filled dams. Consequently, as in any type of construction, data processing techniques to be applied to dam monitoring observations should be able to discriminate between long-term and permanent deformations on one side, and those featuring a cyclical pattern linked to ordinary external forces on the other. For the latter case, the concurrent recording of local meteorological parameters and of the water level in the basin is necessary. In earth-filled dams, water level change does not only reflect the loading of the dam structure but it may also result in fluctuations of the ground settlements due to the lowering and lifting of the water table. 

Traditionally, two different types of absolute and relative displacements may be observed on a dam: horizontal and vertical displacements. On the other hand, Chrzanowski [1] remarks the relevance of considering three-dimensional displacements that may be useful to better analyze deformations generated by concurrent causative reasons. Horizontal movements are usually induced by external forces and may reach the largest magnitude at the dam crest. In the case of concrete dams, thermal and water-level effects may have the major influence on the middle vertical cross-section. For this reason, in many dams, this is the position where a vertical cuniculus is located to host a suspended or inverted plumb-line, able to continuously measure horizontal displacements at different elevations. On the other hand, the pattern of the horizontal displacements may result in deformations only on some portions of the outer downstream. This is a typical case where wider range techniques, such as ADM, may play a key role to detect anomalous and unexpected asymmetrical deformations.

Vertical displacements may be observed on the dam upper crown, in the internal inspection tunnels, in the foundations, and at the interface with the bedrock on the lateral flanks of the valley. Vertical displacements may indicate an overall settlement of the structure if observed with respect to the external area or rotational trends in the case of differential height variation between two points. The need of carefully monitoring the interface between the dam body and the rock boundary is quite important to highlight criticisms linked to relative sinking and shifting, which may be preliminary events to major failures.

Chrzanowski [1] discusses some of the aspects that are important to consider when planning a deformation monitoring scheme, disregarding which are the specific techniques to apply: accuracy, reliability, three-dimensionality of the observations, identification of unstable reference points, automation and continuity, and cost-effectiveness. 

Besides measuring displacements, there are two other relevant aspects in dam monitoring: the conditions of the outer surface of the structural elements and the natural and anthropogenic processes in the surroundings. The alteration of those materials used for preserving the inner strata may lead to some damage of the structural components and may open the way to water infiltration. Therefore, cracks and fractures have to be mapped and monitored to measure extensions and enlargements. Land subsidence (sometimes correlated to the variation of the water level in the basin), slope and rock failures, pore water pressure, water pumping and water infiltration may have an indirect impact on the structural and operational conditions of the dam itself.

## 3. Geodetic and GNSS Sensors for Precise Measurement at Specific Locations

### 3.1. Optical Collimators

**Figure 1:**
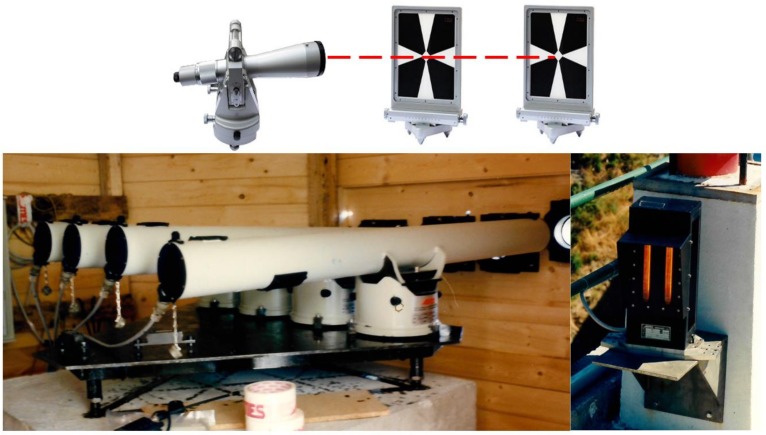
Evolution of collimators from purely manual instruments (on the top) to opto-electronic automatic systems (on the bottom: ISAC 5000 by ISMES S.p.a., Seriate, Italy).

A consolidated technique for monitoring horizontal displacements in large dams is based on optical collimators (Figure 1). They allow for the measurement of horizontal deviations with respect to the optical line-of-sight (LoS) established between a stand-point located at one side of the dam and a portable target that may be deployed along the upper crown of the structure. Such targets can be repeatedly placed on different CPs to investigate multiple alignments. Optical collimators may provide high-precision (at the sub-millimeter or millimeter level) depending on the distance and the local changes of the atmospheric refractive index [1]. However, this is considered as the major weakness of any geodetic method. Since optical collimators are manually operated, they are used for periodic surveying (maximum at daily rate). In principle, an optical collimator may be replaced by a theodolite that may be used for the same purpose, provided it has a similar lens performance.

Automatic collimators based on opto-electronic technology have been developed for continuous dam monitoring and are in operation at several dams. Using such type of sensors, a sub-millimeter accuracy may be reached but they still suffer from local changes of the atmospheric refractive index [31]. A reference target is placed in a stable area on the opposite side of the valley with respect to the collimator station and can be used to mitigate this problem. This solution requires accurate planning of the observation scheme for batteries of automatic collimators. For instance, the LoSs corresponding to the observed and reference targets should not be too far away from one another, since the effect of refraction depends on the square of the distance [1]. LoSs should also be in a upper position with respect to the ground surface and the transit over the water basin should be limited as much as possible.

### 3.2. Geodetic Networks

Geodetic triangulation networks (for 2D horizontal displacements) and optical leveling networks (for vertical displacements) have been the most common approaches adopted for static monitoring of dam displacements in the last century. In the case of geodetic triangulation networks, CPs are usually located on concrete pillars on the dam crest and on stable areas outside the structure to be used as reference points. In correspondence of these locations, theodolites and targets can be set up by using forced centering to eliminate centering errors. An example of such a kind of geodetic networks has been reported by Guler [32]. 

In the last decades, new geodetic sensors have been developed and introduced for routine use in the geodetic monitoring of dams. In particular, robotic total stations (RTS), incorporating a high-precision rangefinder are used for the direct measurement of 3D coordinates of reflectors adopted as CPs. In modern instruments, an internal camera allows for image-based techniques to be exploited for target recognition and precise measurement [33,34], which makes the field operation independent from the operator’s capability and helps speed up data acquisition [35]. Instead of using them in geodetic networks to be periodically re-measured, one or more RTSs may be included into an automatic monitoring system because they allow very short-term repetition of measurements (up to a few tens of minutes, depending on how many CPs have to be targeted). The instrument is installed in a protected hut, from which it periodically measures all the reflectors permanently installed in correspondence of any CP on the dam structure and in the surroundings. The measurement, recording and data broadcasting to a control unit are carried out automatically. As for other optical sensors (see Section 3.1), the effect of local atmospheric refraction on the observation of directions may influence the correct determination of targets, reducing the obtainable accuracy. Range measurements are less affected by changes in local atmospheric conditions. To compensate for the effect of the atmospheric refraction on directions, a subset of CPs positioned on stable areas can be used to update the local calibration model. After such a correction, a millimeter-level accuracy may be reached within a maximum range of 1 km, as demonstrated in Yigit [36]. Here a geodetic triangulation network was applied to detect deformations of a concrete arch dam where periodical and linear horizontal displacements were in the range between 1–10 mm. RTSs integrated to GNSS sensors represent the optimal solutions for monitoring earthen dams and slopes of the water reservoir [1].

Optical leveling is still widely applied for monitoring vertical displacements of large infrastructure [11,37], including dams. Control benchmarks are usually fixed on the outer surfaces of the structure and the stable ground in the vicinity. Benchmarks may also be located in internal inspection tunnels, where they may be fixed on the walls to hang the graduated rods that are necessary for leveling. It should be mentioned that the vertical relative displacements and the distance between two benchmarks may be used to obtain the local rotation of the structure. Optical leveling is still the most reliable method for measuring relative vertical displacements [10], even though it is time-consuming and cannot be operated in a continuous mode. It has the advantage that it does not require the direct visibility between two successive benchmarks but only the presence of an intermediate point from which both can be seen. This makes it possible to work in complex indoor corridors, like inspection tunnels in the dam body, inside the foundations or in rock shoulders. With the use of automatic digital levels, data collection has become more efficient and less dependent on the capability of the operators. Optical leveling provides accurate measurements (precision below 2 mm/km [38]). However, the operations are time-consuming and do not allow for frequent surveying campaigns (usually at a weekly or monthly rate). 

In earthen structures where the expected vertical displacements may be larger than in concrete dams, trigonometric leveling has been applied [39], obtaining a precision of 3.7 mm/km. 

Hydrostatic leveling [40] is a potentially effective alternative for providing continuous and very precise measurements if coupled with devices able to provide automatic continuous readings. On the other hand, such systems require a network of pipes for connecting benchmark points, whose deployment may be difficult and expensive in pre-existing dam structures. 

Alternative solutions for detecting vertical displacements, adopting image processing techniques, have been proposed in the literature [41], but these do not overcome the main limitations of optical leveling. Indeed, a camera has to be placed in proximity to each couple of rods to be measured. Thus, measurement operations are still time-consuming. In addition, some environmental factors, such as the presence of wind, may degrade the achievable precision, as in the case of optical leveling.

### 3.3. Global Navigation Satellite Systems

The use of Global Navigation Satellite System (GNSS) techniques [42] for deformation monitoring of dams has a number of advantages that make them an efficient method in terms of time and cost-effectiveness, compared to other surveying approaches that may provide the same accuracy. First of all, they do not require visibility along the LoS between CPs and reference points, thus allowing the observation of larger areas without restriction on the site locations. Expert operators are only needed for the initial network design and data interpretation, but their presence in the field is not continuously required. Another advantage of GNSS techniques is that they may provide 3D coordinates connected to an absolute and global reference frame, nowadays ITRF2014, last realization of the International Terrestrial Reference Frame [43]. Global or densified regional networks of GNSS geodetic stations, acquiring data continuously, are today available overall the surface of the Earth so that 3D vectors estimated in a local 3D GNSS network can be easily roto-translated in a global reference frame. For example, Drummond [19] and Jiang [20] showed that the increasing diffusion of GNSS Continuously Operating Reference Stations (CORS) may be also exploited for deformation monitoring applications and to establish the external reference.

In order to achieve the highest accuracy, a special attention has to be devoted to the design of the monitoring network. One or more reference stations should be included and regularly controlled using external links in order to determine if movements have occurred. Some statistical techniques have been developed to help with this analysis, see Section 5.2. The GNSS antennas should be mounted using a forced-centering device that guarantees the placement into the exact position occupied in former surveys. Dual-frequency carrier-phase measurements (L1 and L2) have to be used during GNSS data post-processing to improve the atmospheric delays estimations [44]. To reduce errors due to possible signal multipaths, proper site selection to minimize the signal reflection from the surroundings and the use of a suitably designed antenna (e.g., a choke-ring antenna) is recommended. For long-term GNSS campaigns, multipaths may be mitigated during data post-processing, e.g., by sidereal filtering. 

For real-time positioning methods, high acquisition rates (up to 1 s or less) and accurate satellite orbits, as well as clock corrections, are needed. In real-time, GNSS antennas deployed on the dam crest may be dealt with as kinematic rovers relative to the nearby master stations (within a range from 0.1 km to 2 km).

Data processing provides an estimate of the 3D components of vectors between the reference and each CPs (baseline) per each epoch. By analyzing the baseline time series, the deformation pattern may be derived. The reliability of the analysis strongly depends on a proper design of the GNSS control network that should include redundant connections to implement a robust deformation analysis based on a Least-squares adjustment and statistical data analysis and testing [45]. The analysis of deformation detection can be carried out using a variety of test categories as discussed for different GNSS networks by Sacerdote [46]. An investigation into the robust estimate of geodetic networks based on traditional and GNSS observations has been carried out by Nowel [47] and Tasci [48].

One of the most recent and complete studies carried out on dam monitoring by GNSS is the work by Montillet [49] that concerned two large, earth-filled dams: the Hanson and Tolt Dams (Seattle and Tacoma, WA, USA). These structures were monitored using the two main GNSS positioning methods, real-time kinematic (RTK) and static relative post-processing. The RTK positions could be estimated at the centimeter level, allowing for integration into an early-warning system. H24 GNSS data post-processed by standard double-differencing software [50] provided sub-centimeter accuracy that allowed for the detection of long-term deformations.

**Figure 2:**
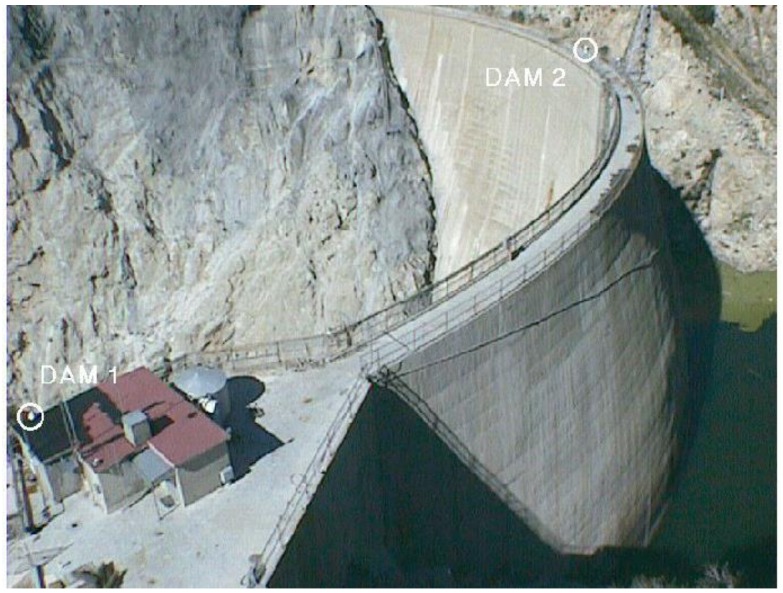
Example of the GNSS (Global Navigation Satellite System) continuous recording stations DAM1 (reference) and DAM2 (CP) at Pacoima dam, USA (image credit: J.A Behr, 1998, https://pasadena.wr.usgs.gov/office/hudnut/SRL/figures/Figure_1.gif).

The potential for an automatic and continuous GNSS monitoring system for dam structural deformation measurement has been already explored in 1989 by De Loach [51]. He proposed the preliminary design of hardware and software for an automated complete system capable of detecting 3D sub-centimetric displacements. The first experiments were carried out in 1995 by Behr [52] on the Pacoima Dam (see Figure 2) and by Whitaker [53] on the Eastside Dam (USA). The former case [54], is one of the first examples demonstrating the feasibility of GNSS dam monitoring based on a permanent network of dual frequency receivers that provided daily coordinates for two years. The pilot study was initially addressed to design a complete system for very accurate measurements of the structure’s response to major earthquakes or other forces potentially affecting the structure over time. Chrzanowski and Szostak-Chrzanowski [55] and Van Cranenbroeck [56] presented other applications of GNSS continuous monitoring systems, the latter paper also including the design of an early-warning system. Cifres [21] and Galan-Martin [22] implemented Kalman filtering to the differential processing of GNSS data to improve the level of precision for dam real-time monitoring.

Rutledge [57] marked an important step by using RTK that is more vulnerable to multipaths and satellite visibility, whilst it cannot be used for dam monitoring when an accuracy of ±1 cm or less is required. In such a case, a method to mitigate the multipath effects consists of the installation of an array of antennas at each station (spatial filtering [58]). 

Several works directly compared deformations obtained from GNSS to deformations obtained with other sensors: coordinatometer [59]; inverted pendulum [57,60]; strain gauges; crack meters and borehole extensometers [60]; pendulum [61]; optical collimators [22]. Liu [62] proved that the application of GNSS networks could take over horizontal optical measurements, while they cannot reach the higher precision granted by optical and hydrostatic leveling. 

In these papers, some problems related to the different reference systems of the various devices were not explicitly addressed. This is probably due to the fact that data gathered by different sensors have never been directly integrated. Dardanelli [63] presented a single roto-translation method for transforming the GNSS coordinates to a local reference frame at the dam’s crown. A deeper investigation devoted to the comparison of data from different devices only once they were in the same reference system is presented by Barzaghi [61]. Yang [64] proposed an interesting application of a pseudolite-augmented GNSS technique that could help overcome some limitations of GNSS-only surveys in unfavorable environments. 

The implementation of new sensors may need the integration of multiple observations requiring a reformulation of the functional model of geodetic/GNSS network adjustment [65].

### 3.4. Terrestrial-Based Radio Frequency Ranging

An interesting innovative approach for the measurement of 3D displacements on specific CPs is offered by terrestrial-based radio frequency ranging technology. This technique relies on a measurement principle that is similar to that of GNSS but based on data broadcasted from ground-based units rather than from satellites. A centimeter-level accuracy for static positioning using a carrier-phase measurement may be obtained for monitoring the structural movement in many applications. For example, the “Locata” system consists of a network (LocataNet) of time-synchronized pseudolite-like transceivers (LocataLites), which can be deployed around a structure to obtain an optimal network geometry despite of the site constraints. In Choudhury and Rizos [66], the first experimental test using “Locata” technology for deformation monitoring of the Tumut Pond Dam (New South Wales, Australia) is presented. This trial was run for 22 hours and yielded millimeter-level horizontal precision and centimeter-level vertical precision for all observed epochs, respectively.

## 4. Remote Sensors for Areal Deformation Measurement (ADM)

ADM techniques [13] offer the opportunity to extend the deformation analysis to larger surfaces rather than to a few CPs placed in some key positions. Indeed, a much larger number of CPs are autonomously selected over the observed region, depending on the adopted technique and the property of the surface in terms of geometry, material and roughness. The outputs are derived from the measured 3D coordinates in the case of terrestrial laser scanning (TLS) or from range and cross-range observations in the case of InSAR sensors. 

For each survey, the geometry of the dam surface, including possibly deteriorated or damaged portions, may be extracted and represented by raster or vector maps. Similarly, the comparison of repeated surveys provides deformation maps or a set of displacement vectors that describe the structural modifications. The ADM techniques that have been experimentally tested on dams include TLS, GBSAR and satellite D-InSAR. Close-range photogrammetry [67] has been also tested but the measurement of dam deformations is strongly limited by the image scale, see Scaioni [68]. Some applications of image-based techniques would be possible for the inspection of the conservation state of surface materials. This option is supported by the use of multi-copter drones, which may fly around the dam body and collect high-resolution images [69]. Another possibility offered by image-based measurement techniques is to analyze the 2D surface displacements in specific areas, for example by means of optical flow/digital correlation techniques [70] or by installing specific targets [71].

### 4.1. Terrestrial Laser Scanning

Terrestrial laser scanning (TLS) can directly provide high-density 3D data and additional information, such as intensity and RGB colors [72,73]. Although its single point measurement accuracy is lower than the one of a total station, change detection methods, may potentially profit from the large data redundancy. Based on these characteristics, the TLS technique yields a challenging and interesting approach to model and analyze possible deformations of objects. The challenge is to identify and, if necessary, parameterize sets of points belonging to the same object in multi-epoch point clouds, since the scanning process cannot repeat the measurements at the same precise locations [13,14,74]. However, deformations in dams are often at the millimeter scale, especially in the case of concrete structures. Consequently, a high data quality is required to try to distinguish real deformations from noise. This task may only be achieved if several aspects related to sensors, data acquisition, processing, and interpretation are carefully considered. From the research on TLS, one may realize that careful measurements need to be planned and systematic errors that may contribute to the error budget have to be investigated. Indeed, if not properly modeled, residual systematic effects may lead to a biased interpretation.

Several systematic error components may contribute to the total error budget of TLS, which are likely to degrade the accuracy of the measurements. In the test field established on the Cancano Dam (Valtellina, Italy) to measure the real performance of TLS for dam monitoring applications [75], the negative effect of multiple systematic errors was highlighted, including instrument inner calibration, incidence angle, registration, surface moisture, solar lighting [76]. On the other hand, for most of them, it seems not possible to come to a complete model. Lindenbergh and Pfeifer [77] demonstrated that the measurement noise of TLS may be reduced to a millimeter level by exploiting data redundancy. 

Several studies have been published on the inner calibration of specific TLS instruments or categories of instruments (e.g., phase-shift and time-of-flight sensors). Different models for correcting inner systematic errors have been summarized in the literature [78]. The influence of laser-beam incidence angle is considered in Soudarissanane [79], providing a way to improve the quality of point clouds. On the basis of these factors, recently Ramos-Alcazar [80] presented a new approach to obtain a complete map-type plot of the theoretical precisions of TLS scanning with time-of-flight method at mid-range distances. 

**Figure 3:**
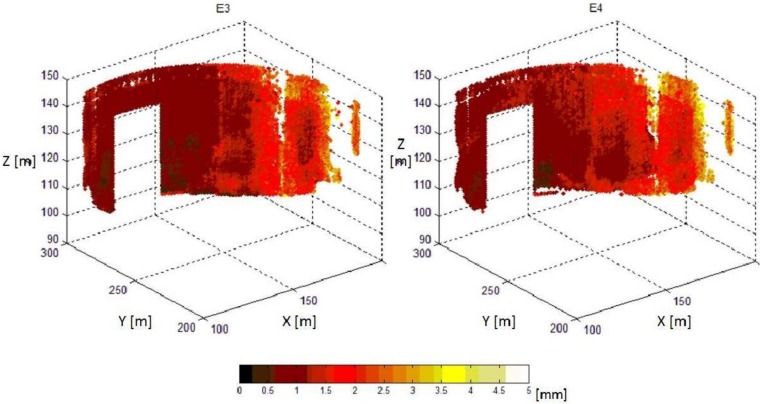
Standard deviations of the final point clouds in a dam [82].

Another important aspect that may result in systematic errors is the registration of multiple point clouds. This task is required to merge more laser scans collected from different standpoints or to co-register point clouds collected at different epochs. In TLS practice, rigid-body transformations are typically applied for registration/georeferencing, since the correct scaling is assumed. In general, corresponding points for registration are defined using targets, whose coordinates may be also independently measured by geodetic techniques to establish a topographic reference system. Very often, retro-reflective targets are adopted to help automatic recognition in scans. In Alba [76], the response of retro-reflecting materials used for such targets was thoroughly analyzed and their employment criticized for applications in dam monitoring. Using artificial targets as a network reference, Eling [81] investigated multi-scans for the monitoring of the Oker Dam at the Harz Mountains (Lower Saxony, Germany). The author showed the presence of residual systematic errors with a magnitude of approximately 5 mm that should be further investigated. Inspired by this, external error modeling in a combined registration model and fine registration were investigated to decrease the size of systematic errors as well as to improve the precision of the final point clouds [82], see Figure 3. This extended methodology could reduce systematic errors by approximately 3 mm, along with a reduction of a posteriori variance by approximately three times. In addition, the consistent series of experiments presented in Wunderlich [83] deserves to be mentioned, since some objective criteria to evaluate the accuracy, geometric truth, optimal measurement speed, and realistic maximum operational range of TLS were studied.

Modeled TLS data may provide an accuracy higher than the single point precision [84]. Different models may be used independently from the specific application [85]. A parametric surface model suitable for approximating a point cloud for monitoring purposes may depend on the shape of the object under investigation. When possible, the outer surface of the dam may be globally interpolated using a simple shape, which is controlled by a set of parameters. The estimated parameters at each epoch are then compared to determine whether the object has undergone deformations [86,87]. Statistical testing is applied to check if deformations are significant [2].

Alternatively, the whole point cloud describing the dam surface may be segmented into several subsets, each of them to be approximated by using simple shapes (piecewise surface models). Planes [77,78] and rotational paraboloids [88] have been used for this purpose. Schäfer [89] adopted a Delaunay-triangulation method for calculating a uniform and regular grid that is identical in each point cloud to compare. Similar approaches were developed by Tsakiri [90].

### 4.2. Ground-Based InSAR

Ground-based synthetic aperture radar (GBSAR [15]) is a radar-based terrestrial remote-sensing technique used to measure and monitor deformations. It consists of a radar that emits and receives microwaves, repeating this operation by moving along a rail track. The GBSAR imaging capability is based on the synthetic aperture radar (SAR) technique [91]. The sensor incorporates a coherent radar system, which measures the amplitude and the phase of the received radar signal. The phase, which brings the geometric information related to the deformation of the observed scene, is exploited by using the interferometric technique. 

In the literature, two different types of acquisition modes are described: the continuous and discontinuous modes. In the case of the continuous mode, the GBSAR is permanently installed in front of a given structure, acquiring data periodically. The acquisition period can be as short as a few minutes, or a few seconds, depending on the characteristics of the adopted radar instrument. This configuration is suitable to carry out near real-time deformation monitoring, while it offers the best performances in terms of measurement density, precision and robustness [15]. Examples of continuous dam monitoring are described by Alba [92], Luzi [93], and Talich [94]. In the discontinuous mode, the GBSAR instrument is installed and dismounted at each acquisition campaign, revisiting the site periodically, e.g., monthly or twice a year. Examples of such an acquisition mode are described by Tarchi [95], Jenkins [96], Di Pasquale [97], and Mascolo [98].

**Figure 4:**
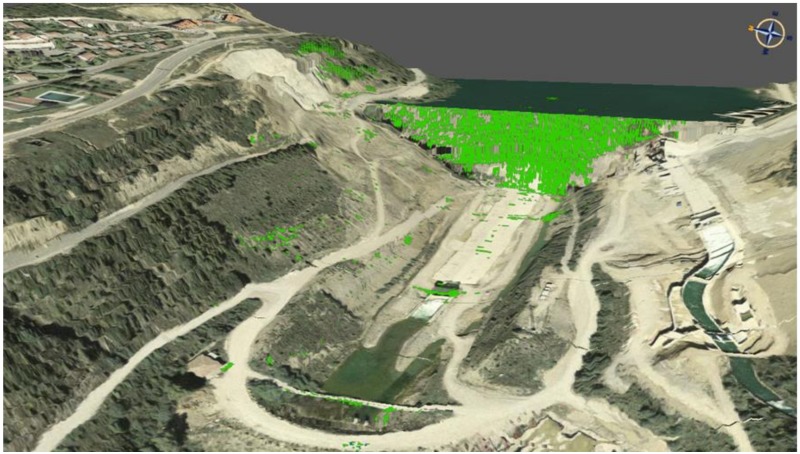
Example of the dense spatial sampling obtained by GBSAR in the monitoring of a dam (green points are observed points). Image credit to M. Crosetto (CTTC/CERCA, Castelldefels, Spain).

GBSAR-based deformation monitoring offers interesting characteristics that make this solution complementary to other techniques. The first advantage is related to the remote-sensing nature of GBSAR, which can be accomplished without installing sensors or targets on the structure at hands, while operating at a significant distance from it (up to 1–2 km). This can be useful to guarantee the safety of the monitoring activities, especially in the case of emergency situations [99]. The second remarkable advantage is that GBSAR may be operated day and night and in all weather conditions. A third advantage is its capability to provide a dense 2D spatial sampling of a given scene, as shown in the example in Figure 4. This is an important advantage with respect to the point-wise deformation measurement techniques based on CPs because it may provide a more complete and detailed picture of the deformation pattern at hand. Regarding the spatial sampling density, this depends on the sensor distance from the dam and on the relative geometry between the sensor and the structure. At short ranges and under favorable conditions, GBSAR may provide a few measurements per square meter. This is substantially lower than the density that can be obtained from TLS. This aspect is mitigated by the fourth GBSAR’s advantage: the high precision of its measurements, ranging from ±1 mm to sub-millimeter. This has been demonstrated in different validation studies based on data recorded by means of traditional sensors, typically installed in the dam structure, see Tarchi [95] and Alba [92]. It is worth emphasizing that the precision depends on different factors, the most important of which is the *coherence* of the measurements. In the case of dam monitoring the coherence is usually high; however, it is higher in the continuous mode than in the discontinuous one. For this reason, the best measurement performance is achieved in the continuous mode. A fifth advantage is that the GBSAR monitoring provides a high temporal resolution: image acquisition can be operated in matters of a few minutes, if not a few seconds [15]. This property can be exploited to perform near real-time deformation monitoring. A sixth advantage is that the procedure to carry out GBSAR measurements is relatively simple; it can be performed by non-skilled operators as well.

The most important limitations of GBSAR are briefly described in the following. First of all, data coherence is a condition sine qua non to carry out monitoring, as already discussed. The second limitation concerns the ambiguous nature of the GBSAR deformation measurements because they also require the estimation of the phase ambiguity. This operation is usually straightforward when using continuous-mode data, while it can be critical when using the discontinuous measurements [100]. Another intrinsic GBSAR limitation is the mono-dimensional nature of the observed deformations; given a 3D displacement, the GBSAR measures its projection along the radar LoS. Those displacements that are perpendicular to the LoS cannot be seen by GBSAR. The interpretation of the LoS measurements is usually done by making assumptions on the geometry of the deformations at hands. A fourth limitation is due to the atmospheric effects, which are mainly related to the variation of the relative humidity of the atmospheric conditions between the sensor and the target during data acquisition. The atmospheric effects are stronger at long sensor-to-target distances and at large dam dimensions. A work devoted to the analysis of the atmospheric effects in a dam study is described by Xing [101]. Other works related to the atmospheric effects include Luzi [102], Rödelsperger [103], Iannini and Guarnieri [104], and Iglesias [105]. A final limitation, which needs to be carefully considered to achieve a correct interpretation of GBSAR measurements, is due to the fact that some radar data may correspond to various parts of the same dam (this can be due to multiple reflections). In addition, some of the measurements can be related to some loose parts, e.g., wiring, railing or lamps. Examples of data interpretation of GBSAR measurements of a complex dam structure are described by Qiu [106] and Huang [107].

A number of experimental GBSAR monitoring results show the potential for dam deformation monitoring. However, it is worth noting that an operational GBSAR dam monitoring system, for instance, based on a permanent installation, has not been documented yet in the literature. This is probably due to different causes, among which is the high cost of the instrumentation.

Real-aperture radar interferometric sensors may represent an alternative to GBSAR [108]. However, the major problem with this type of sensors consists in the ambiguous identification of the observed targets without the installation of artificial corner reflectors, since they have only 1-D range resolution. 

### 4.3. Spaceborne Advanced DInSAR

Spaceborne DInSAR was initially applied to the analysis of ground subsidence over large areas or to detect earthquake-induced deformations [109]. Thanks to the improvement of the sensor spatial resolution and data processing techniques, it has been extended to applications at more local scale, like unstable slopes and large structures. Recently, thanks to a few-meter ground resolution of SAR images and the reduced repeat-pass time, advanced differential InSAR (A-DInSAR) techniques extended their applicability for measuring deformations of civil engineering structures as well. Several applications to dams have been reported in the literature. The use of A-DInSAR cannot be finalized to set up continuous monitoring and early-warning because the repeat-pass time of satellites (a few days) is still too long. However, A-DInSAR has been widely used to measure the deformations of dams over limited time periods or to reconstruct the past failures of some reservoirs on the basis of archive SAR data [18,110]. In addition, the same SAR images adopted for measuring deformations of the dam structure and the hydraulic infrastructures could also be exploited for assessing the stability of the slopes at the border of the water basin [111,112,113,114]. Since SAR images generally cover wide areas, their use is recommended when the analyzed infrastructure spans over several kilometers. This is the case for large basins bordering communication corridors as reported by Michaud [115]. Thanks to the wide-range potential, A-DInSAR can be used to detect subsidence in areas where dams are located, since such problems might have an influence on the stability of dams themselves (see, e.g., Fergason [116]).

Among the A-DInSAR techniques [117], Persistent Scatterer Interferometry (PSI) has gained great popularity for deformation measurement of man-made structures. In Crosetto [118] a review of PSI and a presentation of existing implementations can be found. Lazecky [119] reported three examples of PSI applications to monitor deformations of three different types of dams, using SAR data sources: the Charvak Dam in Uzbekistan based on ENVISAT-ASAR data, the Three Gorges Dam in China based on Cosmo-SkyMed data, and the Plover Cove Dam in Hong Kong based on TerraSAR-X data (see also Lazecky [120]).

The application of A-DInSAR was used to detect surface displacements of an earth-filled dam at La Pedrera Reservoir (Alicante, Spain [113]). The open geometry of such kind of barrages, facilitating the illumination from spaceborne SAR sensors, allowed the detection of a displacement of about 13 cm along the satellite LoS between August 1995 and May 2010. A data set composed of medium resolution ERS-1, ERS-2 and Envisat-ASAR images was mainly used, whilst a small test with high-resolution TerraSAR-X data was operated over a couple of years. The joint analysis of historical instrument surveys and A-DInSAR-derived data allowed the identification of a long-term deformation pattern. This study demonstrates the integration of A-DInSAR with in-situ techniques, which helps provide a complete spatial picture of the displacements in the dam thereby helping differentiate the causal mechanisms. Earth-filled dams were also investigated by Honda [121] using ALOS PALSAR data and by Di Martire [122] using a series of Envisat-ASAR images. In the latter study, a comparison with independent in-situ measurements showed an agreement below 1 cm. In Vöge [123], the dependency of the quality of results obtained from A-DInSAR and the geometry of the illuminated surfaces is also confirmed.

**Figure 5:**
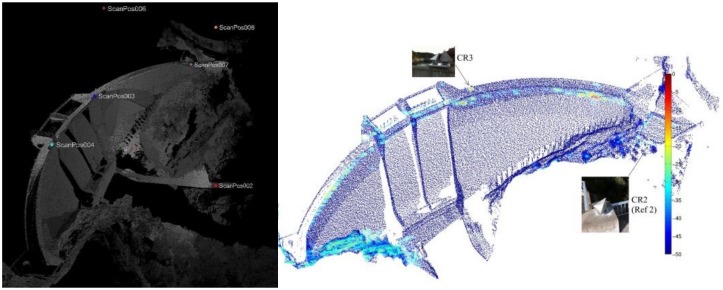
Integration of a point cloud from TLS surveying and results from DInSAR analysis with the aim of a better visualization and localization of displacement vectors (image credit: Anghel 2016 [114]).

Anghel [114] applied TLS, GNSS and theodolites for precise 3D modeling of the Puylaurent concrete dam in France. The model was then used for the projection of deformation components from A-DInSAR processing to obtain a more realistic interpretation and analysis of vector point displacements, as shown in Figure 5. The analysis, carried out over a time span of approximately eight months, demonstrated a good agreement between results from A-DInSAR and in-situ measurements. 

The validation of A-DInSAR has received a lot of attention in the last two decades [118]. In addition to the examples already discussed, some others include a comparison with in-situ geodetic measurements [124,125], leveling data [126], and a comparison with a network of ground-based sensors [23,122]. However, the observations from space cannot ever be trusted as a unique data source for the measurement of surface displacements but need to be integrated and validated with respect to geodetic/GNSS data records. 

The results obtained so far are quite encouraging for promoting future efforts in the application of spaceborne SAR data for dam deformation measurement. Indeed, the availability of last-generation very high-resolution images acquired by TerraSAR-X and Cosmo-SkyMed constellations may allow us to obtain better ground resolution and shorter revisiting-time by means of algorithms that may produce increasingly reliable results, see Osmanoğlu [127].

## 5. Integrated Monitoring Systems, Data Processing and Methods for Deformation Analysis

### 5.1. Integrated Monitoring Systems

Due to the presence of several processes that might affect the dam stability and because of the size of such structures, monitoring is often quite complex and may require an integrated monitoring system (IMS [1]). While in the past this term only referred to the use of multiple sensors to collect different types of observations related to the same dam (see Giussani [128]), today IMSs may incorporate sensors that concurrently collect data in an autonomous and continuous way. Consequently, the communication among sensors plays an important function in an IMS. On one side, the technological infrastructure for data transfer has been largely investigated and many solutions proposed. Wireless technology has been largely exploited [129], even in the case of hydropower plants, where data safety, reliability and confidentiality have a primary relevance. These solutions may have some limitations from technical and/or legal point of views. On the other side, several protocols for smart-sensor communication and interoperability, also through web-services, have been and will be further developed [130].

Examples of IMSs applied to dam monitoring are difficult to find in the literature. This is mainly motivated by the fact these applications have not been given too much attention by the scientific community, though they are really important in the daily practice of those organizations dealing with dam administration and management. An interesting example is the system GOCA (www.goca.info) developed at Hochschule Karlsruhe (Karlsruhe, Germany), which integrates GNSS, theodolites and other sensors on the hardware side, along with models for data analysis and prediction. The GOCA system can be used for online control and early-warning. A few software packages may be used to integrate observations from multiple permanent geodetic sensors. For example, the ALERT system developed at the Canadian Centre for Geodetic Engineering [131] can be used to connect several RTSs and GNSS sensors. 

Another interesting aspect of sensor/data integration consists of the corroborative use of data from different sensors to improve their potential and efficiency. In the previous sections, some experiences have been already reported. Among these, a clear example is found in Anghel [114], who combined spaceborne SAR image processing with a 3D model of the dam obtained from TLS surveying. The availability of such a detailed model is exploited to better define the spatial geometry of the radar signal (see Figure 5). In this way, an augmented interpretation of 3D displacements can be achieved. In Mascolo [97] and Nico [132], the integration of GBSAR and a spaceborne A-DInSAR analysis of Cosmo-SkyMed data was exploited for studying horizontal and vertical displacements of old embankment dams. The former instrument was adopted for measuring horizontal displacement vectors, the latter for vertical displacement vectors. Guedes and da Silva [38] presented the analysis of a comparative study including the use of geodetic measurements, plumb-line and GNSS. In Alcay [133], the integration of a pendulum in the vertical cross-section of the dam and geodetic observations revealed either periodical and linear trends due to seasonal temperature oscillation and water level change in the reservoir.

The ground displacement measurement of dams should always be complemented by observations in borings to assess the geotechnical conditions in the subsurface. Borings may be operated by applying standard penetration tests or cone penetration tests. Alternatively, geophysical investigations may be applied [134]. This aspect is particularly important for the stability in earth-filled dams, in order to mitigate the risk of a slope failure. Indeed, while horizontal and vertical displacements could be measured on the outer surface by using geodetic/GNSS techniques, horizontal and vertical stresses should be measured at some locations in boreholes together with pore water pressure. The combination of both stresses and the infiltrated water may influence the dynamic soil response and the liquefaction resistance [135,136]. Deeper studies on the subsurface conditions should be organized in seismic areas to evaluate the actual soil behavior and local site amplification effects [137].

### 5.2. Conventional Deformation Analysis

The data collected by such a variety of sensors that can observe different parameters on multiple portions of the same dam are prone to be integrated to better analyze the dam deformation trend. At this stage, one important topic is the methodology to apply for the conventional deformation analysis (CDA [138]). This consists of a comparison between the coordinates of CPs and reference points included in a geodetic network between observation epochs. In general, statistical testing [45] is applied to detect significant changes in the point locations to be interpreted as CP displacements. The critical aspect is that “real” displacements and measurement noise are in general very close and the stability of reference points may not be guaranteed [139]. In general, the analysis is developed through three main steps [36]: (1) free-net adjustment and outlier rejection at each epoch; (2) a global congruency test to detect the presence of significant changes (i.e., potential displacements); (3) localization procedures. The latter consists on the identification of CP and reference point displacements. Different statistical techniques have been applied to this aim. Several approaches have been based on S-transformations [36,140,141,142,143]. Other methods are based on the relative confidence ellipse method [144] and the implicit hypothesis method [145]. 

### 5.3. Time-Series Analysis for Investigation of Deformations’ Causative Reasons

Time series of displacements derived from geodetic and remote-sensing observations as well as geotechnical data collected on dams have to be analyzed in order to find the relationships with loads. These are mainly the water level in the reservoir and the external temperature. Once these relationships have been identified, from the knowledge of loads, short- (a few months) and long-term (one year) forecasting of dam deformations may be carried out. 

The complexity of this analysis depends on several factors: the type of dam, the specific phase of its lifetime (e.g., construction, first filling, operation, refurbishment), the age of the structure, the presence of structural or deterioration problems. In addition, each causative reason may feature different patterns: harmonic fluctuations, locally linear trends, irregular patterns. As a result, investigating the dependency of displacements upon loads may be quite difficult. Standard statistical analysis and filtering techniques may not be sufficient to capture deformation trends excluding seasonal effects. In order to improve this task, some tailored methods have been developed and experimentally tested. These methods could be classified as statistical methods (see Section 5.3.1) and deterministic methods (see Section 5.3.2) [1].

#### 5.3.1. Statistical Methods

The statistical methods try to establish a relationship between forcing actions and the resulting deformations measured on the dam without considering the mechanical aspects. This type of analysis may provide an important contribution to dam deformation analysis and interpretation when long time-series are available. Statistical methods can also precede the application of deterministic methods in a cascaded processing workflow.

Multi-regression models (MRT) have a long tradition in the analysis of dam deformations, as reviewed in Zou [146]. However, they require to precisely define those functions linking deformations to loads. Since external temperature and hydrostatic pressure may have seasonal characteristics, the effectiveness of MRT to forecast dam behavior is limited to short periods (a few months). In order to overcome this limitation, in Mata [147], a comparison between MRT and neural networks (NN) is reported. Though NN have shown better results for predicting deformations within a few months, both methods may be used in parallel to enforce the reliability of the deformation analysis. Zou [146] also compared MRT with back propagation NN (BPNN) and the seasonal integrated autoregressive moving average (SARIMA) model within the application to the Hoa Binh Dam in Vietnam. They found that the MRT and SARIMA models may be useful to forecast deformations up to four months, while for a longer time (up to one year) the combination of the SARIMA and BPNN models provided better results. 

Dai [148] proposed an independent component analysis (ICA) method for modeling different signal components, which were assumed to be mutually independent.

Yigit [36] applied a correlation analysis to the displacements observed from a geodetic network during the first filling of the Ermenek Dam (Karaman province, Turkey). They found a correlation between the periodic deformation pattern and the seasonal variation of external temperature, while a linear deformation trend was related to the increase of the water level during filling. 

Pytharouli and Stiros [27] considered the relation between the water level in the basin and displacements at the crest of the Ladon concrete dam (Arcadia, Greece), where long-term observations were available (more than 30 years). Since no apparent linear correlation was shown, they applied three techniques for spectral analysis, which revealed the same periodicity in the fluctuation of both data sets. This demonstrated the causative dependency of dam deformation and hydraulic load. The same authors applied a threshold correlation method to the analysis of crest displacements of the Kremasta earthen dam in Greece [37]. A long-term series of observations spanning over 35 years was available also in this case study. They found a combined effect of three parameters (water level, monthly increase rate of water level, and rainfall rate) on dam displacements but only when all of them concurrently overcame some individual critical thresholds. 

#### 5.3.2. Deterministic Methods

The deterministic methods compare displacements and geotechnical measurements that have been observed on a dam to the corresponding parameters obtained from numerical modeling of the structural behavior under external loads [149]. This integration plays a key role in the study of those processes that occur in dams during the construction and post-construction phases. A good agreement between the forecasted and measured deformation demonstrates that the structure is working according to its design. On the other hand, the observation of possible departures may offer some suggestions for new forthcoming projects. In Hariri-Ardebili [150], a list of numerical modeling techniques adopted for dams is reported, being the finite-element method (FEM) and its derivatives one of the most popular approaches due to the capability of handling complex geometries as well as to adapt to specific geological and boundary conditions [39]. Numerical models are based on the knowledge of input loads, material properties and physical laws controlling the stress-strain relationship [1].

Deterministic models of expected deformations may be used for the design of a new monitoring scheme or for the improvement of existing ones. Szostak-Chrzanowski [151] presented an application of numerical modelling to design the monitoring scheme for the Shuibuya concrete face rock-filled dam in China.

Chen [141] developed a generalized method for the geometric analysis to determine displacements and strain field of a deformable body in space/time domain, which is based on geodetic and geotechnical measurements at specific locations. An example of such an application is reported in Chrzanowski [152]. 

Gikas and Sakellariou [39] analyzed the settlement behavior of the Mornos earth dam (Lidoriki, Greece) over more than 30 years, which included construction, first filling and most of the operational time up until publication (2008). The results from a numerical back analysis based on FEM have been compared to vertical displacement observations obtained from geodetic leveling data measured on the dam crest and along the main inspection tunnel. An average agreement of 3 cm was found, showing the correctness of the FEM set up in terms of input loads and geotechnical conditions. In the study already reviewed in Section 5.3.1, Yigit [36] found a good agreement between the observed geodetic deformations and the ones predicted from the FEM of the structure during first filling. Acosta [153] compared vertical settlements from high-precision leveling and horizontal displacements from GNSS to the outputs of FEM in the case of the Arenoso earth dam (Andalucia, Spain). Some average differences of 20 cm for vertical displacements and 6 cm for horizontal displacements at the crest were found, respectively. These discrepancies were probably motivated by the simplification assumed during the application of FEM. 

**Figure 6:**
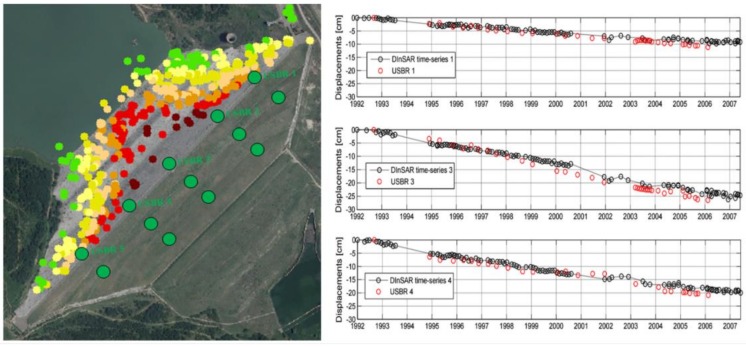
On the left, persistent scatterers extracted by using A-DInSAR techniques at the Genzano di Lucania earth dam (Italy) [23]. The colour of each point indicates the vertical velocity (from green to red). On the right side, the time series (1992–2007) of vertical displacements in correspondence of three persistent scatterers selected on the dam crest in the proximity of three topographic benchmarks (green circles with black contour). The acronym USBR stands for United States Bureau of Reclamation.

A further step forward in this research direction was made by Corsetti [23], who recently presented the application of an A-DInSAR technique to generate deformation time series at a full spatial resolution and from multi-sensor SAR data. The target of this project was to measure the vertical consolidation displacement of the Genzano di Lucania earth dam (Genzano di Lucania, Italy). As shown in Figure 6, a large number of observed persistent scatterers were distributed along the whole structure and were characterized by millimetric accuracy on the displacement rates. These points have been successfully adopted for the calibration of numerical models to simulate the structural behavior of the dam under stress conditions. 

While several papers have been applied to demonstrate how well the real dam have followed the design conditions, the progress of research seems to go in the direction of combining observations and numerical modeling. This solution may be useful to achieve a better calibration of theoretical numerical models according to the real observed conditions, offering the chance to carry out more precise and timely forecast of possible critical conditions.

## 6. Conclusions

This paper has reported a review of modern geodetic, GNSS and remote-sensing techniques that may be applied for monitoring dam deformations, together with geotechnical and structural sensors. While geodetic techniques have a long tradition and may benefit today from the up-to-date instrument technology and degree-of-automation, the other methods represent the real novelty and potentially open unprecedented perspectives. For this reason, a thorough analysis has been done of the so-called areal deformation measurement (ADM) techniques, including ground-based (terrestrial laser scanning and ground-based InSAR) and spaceborne (DInSAR) sensors. Since ADM techniques allow the extension of the investigated area beyond the limited locations defined by control points. GNSS-based methods may offer now a lower dependency on the local topographic constraints (i.e., the limited line-of-sight between sensors), provided that there is sky visibility. Section 5 has also given an overview about sensor and data integration, data processing techniques, and on statistical and deterministic methods to interpret and predict dam deformations.

The general picture offered by these new technologies is promising, even though future efforts should be put to understand which techniques have the potential to follow-up in the regular practice of dam monitoring and surveillance and which may only offer a contribution to scientific investigations. Attention should be also given to other emerging technologies, such as synthetic aperture LADAR [154]. 

The increasing use of satellite Earth observations (EO) is an important point to discuss. On one side, such data may be used for the remote monitoring of dams and the nearby environment, whose influence on the safety of the barrage itself may be really relevant. On the other side, the growing availability of new improved data sets (for example, Sentinel data) is supposed to foster even more the application of satellite EO data.

Attention should be also paid to the integration of data coming from different types of sensors that may offer a different prospect of the same construction. In particular, geodetic measurements can provide surface horizontal/vertical displacements of control points located in key positions, remote-sensing techniques may output a broader picture of displacements over the full structure and the surrounding, while geotechnical/structural sensors may yield important information of those processes inside the dam structure and the foundations. Such a data/sensor integration may create added value and increase data redundancy to be used for cross-checking observations.
